# Effectiveness of Weight-Bearing Ratio with Berg Balance Scale in Geriatric Patients Undergoing Recovery-Phase Rehabilitation: A Pilot Study

**DOI:** 10.7759/cureus.77939

**Published:** 2025-01-24

**Authors:** Takuhei Ozeki, Tomonori Tanabe, Yuta Yamane, Kouhei Miyata, Makiba Nakai, Ririko Mukai, Akio Goda, Yoshinori Maki

**Affiliations:** 1 Rehabilitation, Hikone Chuo Hospital, Hikone, JPN; 2 Physical Therapy, Faculty of Health and Medical Sciences, Hokuriku University, Kanazawa, JPN; 3 Neurosurgery, Hikone Chuo Hospital, Hikone, JPN

**Keywords:** ambulation, berg balance scale, elderly, recovery phase, rehabilitation, weight bearing ratio

## Abstract

Introduction: Independent ambulation is crucial for elderly patients in recovery-phase rehabilitation. However, a simple clinical indicator linking balance ability to safe ambulation remains elusive. This study aimed to evaluate the utility of weight-bearing ratio (WBR) as a simple measure of ambulation ability in elderly patients undergoing recovery-phase rehabilitation.

Methods: 35 patients (male:female=11:24) with a Functional Ambulation Category (FAC) of 3 or higher were enrolled. Data included age, sex, weight, Mini-Mental State Examination (MMSE) scores, Berg Balance Scale (BBS) scores, and WBR. Patients were grouped by ambulation aid: walkers/cars (Group A) and sticks (Group B). Group differences were analyzed statistically (p<0.05).

Results: Groups A and B averaged 85.1 ± 6.4 and 77.4 ± 6.4 years, respectively. BBS scores and WBR differed significantly between groups (p<0.05). A strong positive correlation existed between BBS scores and WBR (r=0.824, p<0.01), persisting after controlling for age (r_p_ =0.781, p<0.01).

Conclusion: Our results showed a significant and positive correlation between BBS scores and WBRs of elderly patients undergoing recovery-phase rehabilitation who were evaluated according to ambulation aid. WBR may be a simple and useful measure of ambulation status in those patients.

## Introduction

In the aging Japanese population, recovery-phase rehabilitation is crucial for improving functional outcomes after cerebrovascular disease or motor disorders [1]. Regaining independent ambulation without falls is a primary goal of this rehabilitation. However, age-related muscle weakness often hinders the achievement of complete ambulation independence. Many elderly patients require bilateral upper extremity support (walkers, wheeled mobility aids) initially, progressing to unilateral support (canes) as their abilities improve [2]. The selection of appropriate ambulation assistive devices currently relies heavily on the subjective judgment of physical therapists. A simple, objective assessment of balance to guide this decision-making process is lacking.

The Berg Balance Scale (BBS) is a widely used and validated assessment of balance in elderly individuals, effectively predicting fall risk [3-5]. Because this scale consists of 14 variables measuring balance performance, its administration is time-consuming (approximately 20-30 minutes), limiting its practicality for routine clinical use. Therefore, a simple and more efficient measurement to evaluate the balance ability of elderly patients seems ideal.

Weight-bearing ratio (WBR), a simpler and more efficient measure of balance and ambulation, has shown promise in evaluating balance abilities [6,7]. This scale is also reported with the correlation of walking and climbing stairs in elderly or post-stroke patients [8]. The correlation between WBR of healthy volunteers in the sitting position and knee extensor muscle strength was also described by Moriyama et al. [8]. Despite its potential, research on the WBR in elderly patients during recovery-phase rehabilitation is limited.

This study aimed to compare BBS scores and WBR in elderly patients undergoing recovery-phase rehabilitation, investigating the correlation between these measures and evaluating WBR as a potential tool for selecting appropriate ambulation support. Furthermore, the study investigated the relationship between age and these parameters.

## Materials and methods

Study design

This retrospective cohort study analyzed data from hospitalized elderly patients who received rehabilitation in the recovery phase ward of Hikone Chuo Hospital between December 2022 and July 2023. This study was approved by the ethical committee of Hikone Chuo Hospital (approval number: 2023-4). Although we have subacute, recovery, and chronic-phase wards in our hospital, we thought that elderly patients undergoing recovery-phase rehabilitation should be favorable to evaluate their ambulation ability because of their relatively stable status and improved or at least maintained their activities of daily living (ADL). The number of recruited patients could be a limitation of this study; however, the selected patients seemed appropriate. 

Participants

Patients were included if their ambulation status, at admission or during admission, corresponded to a Functional Ambulation Category (FAC) of 3 or higher (FAC 3: requiring supervision and verbal cues; FAC 4: independent ambulation on level surfaces but requiring supervision for negotiating obstacles; FAC 5: completely independent ambulation) [9,10]. To minimize individual bias, multiple therapists evaluated patients' ambulation status. Patients categorized into FAC 0-2 were not included. Patients were excluded if they were unable to follow rehabilitation instructions due to cognitive impairment or if their clinical data were incomplete. Those patients whose clinical status was aggravated because of worsening underlying disease or unexpected comorbidities were excluded, either. This study was approved by the Hikone Chuo Hospital Ethics Committee (approval date: July 21st, 2023). Written informed consent was obtained from all participants and their families. Potential risks of falls and efforts to minimize accidents were conveyed to participants and their families before the initiation of the study. Participants and their families understood the risks and agreed to participate in this study.

Data collection

The following baseline characteristics were collected from medical records: sex, age, body weight, and the underlying medical condition requiring rehabilitation. Cognitive function was assessed using the Mini-Mental State Examination (MMSE) upon admission. Balance ability was evaluated using the BBS and the WBR.

Berg Balance Scale

The BBS assesses balance through 14 tasks scored from 0 to 4, yielding a total score ranging from 0 to 56 [3,11,12]. Higher scores indicate better balance. This scale is reported with good reliability and validity in elderly population [13].

Sitting to Standing

Patients were indicated to stand from a sitting position. A score of 0 was assigned to requiring moderate or severe assistance in standing up, 1 to requiring mild assistance in balancing or standing up, 2 to independently standing up with manual support after several trials, 3 to independently standing up using hands, and 4 to independently standing up without any support.

Standing Unsupported

Patients were indicated to stand still without any support for two minutes. A score of 0 was assigned to being unable to stand without any assistance for 30 seconds, 1 to being able to stand with assistance for 30 seconds, 2 to being able to stand without assistance for 30 seconds, 3 to being able to stand for two minutes under supervision, and 4 to being able to stably stand for two minutes without supervision.

Sitting Unsupported

Patients were indicated to sit still without hand support or leaning back. A score of 0 was assigned to being unable to sit without any assistance for 10 seconds, 1 to being able to sit for 10 seconds, 2 to being able to sit for 30 seconds, 3 to being able to sit for two minutes under supervision, and 4 to being able to stably sit for two minutes without supervision.

Standing to Sitting

Patients were indicated to sit from a standing position. A score of 0 was assigned to requiring assistance in sitting, 1 to independently sitting but being unable to be stably seated, 2 to sitting with the dorsal surface of the lower extremities leaning on a chair, 3 to sitting using hands, and 4 to sitting without using hands.

Transfers

Patients sitting in a chair or wheelchair were indicated to transfer to a bed and back to the initial position from the bed. A score of 0 was assigned to requiring assistance of two persons in safely transferring, 1 to requiring assistance of one person in transferring, 2 to being able to transfer under oral indications or supervision, 3 to being able to transfer with hand support, and 4 to being able to transfer with slight hand support.

Standing with Eyes Closed

Patients were indicated to stand still with their eyes closed for 10 seconds. A score of 0 was assigned to requiring assistance in avoiding falls, 1 to being unable to stand with their eyes closed for three seconds but able to stand still, 2 to being able to keep the position for three seconds, 3 to being able to keep the position for 10 seconds under supervision, and 4 to being able to stably keep the position for 10 seconds.

Standing with Feet Together

Patients were indicated to stand with their feet together without any support. A score of 0 was assigned to requiring assistance in standing with their feet together and unable to keep the position for 15 seconds, 1 to requiring assistance in standing with their feet together but able to keep the position for 15 seconds, 2 to being able to stand with their feet together but unable to hold the position for 30 seconds, 3 to being able to stand with their feet together for a minute under supervision, and 4 to being able to independently stand with their feet together for a minute.

Reaching Forward While Standing

Patients in standing positions were indicated to raise and hold an arm horizontally. Thereafter, they were also indicated to reach the arm forward as possible without rotating the trunk. The maximum distance of the reach was measured. A score of 0 was assigned to requiring assistance in holding the posture when reaching the arm forward, 1 to being able to reach forward under supervision, 2 to being able to reach 5 cm forward, 3 being able to reach 12 cm forward, and 4 to being able to reach 25 cm forward.

Retrieving an Object from the Floor

Patients in standing positions were indicated to retrieve an object on the floor placed in front of their legs. A score of 0 was assigned to requiring assistance in holding the posture when attempting the task, 1 to being unable to retrieve the object and requiring supervision in attempting the task, 2 to being unable to retrieve the object but able to reach the arm 2-5 cm forward, 3 to being able to retrieve the object but requiring supervision, and 4 to being able to easily and safely retrieve the object.

Turning Trunk (Feet Fixed)

Patients were indicated to rotate their trunks to the right and left behind, respectively. A score of 0 was assigned to requiring assistance in balancing and avoiding falls, 1 to requiring supervision in attempting the task, 2 to being able to rotate the trunk to 90 degrees holding the balance, 3 to being able to perform the task in a unilateral side but unable to smoothly shift their weight to the other unilateral side, and 4 to being able to perform the task in the bilateral sides.

Turning 360 Degrees

Patients in standing positions were indicated to turn 360 degrees twice, from right to left and vice versa. A score of 0 was assigned to requiring assistance in turning, 1 to requiring oral indications or supervision in attempting the task, 2 to being able to slowly turn 360 degrees, 3 to being able to turn 360 degrees only once within four seconds, and 4 to being able to perform the complete task within four seconds.

Stool Stepping

Patients in standing positions were indicated to put their feet on a step stool (height: approximately 12-20cm) in front. The patients tried to perform eight times in total (right and left feet: four times, respectively). A score of 0 was assigned to requiring assistance in avoiding falls or unable to perform the task, 1 to requiring assistance in performing the task three times, 2 to being able to perform the task four times under supervision, 3 to being able to independently perform the task eight times for more than 20 seconds, and 4 to being able to perform the complete task within 20 seconds.

Tandem Standing

Patients were indicated to hold tandem standing posture as possible. A score of 0 was assigned to being unable to step forward because of losing the balance, 1 to requiring assistance in stepping forward but able to hold the posture for 15 seconds, 2 to being able to independently step forward (a narrow step) and hold the posture for 30 seconds, 3 to being able to independently step forward (a wide step, not enough as tandem standing) and hold the posture for 30 seconds, and 4 to being able to independently tandem stand and hold the posture for 30 seconds.

Standing on One Leg

Patients were indicated to stand on one leg as long as possible. This task was repeated twice on the right and left legs. A score of 0 was assigned to requiring assistance in avoiding falls or unable to perform the task, 1 to being able to independently stand on one leg and hold the posture for less than three seconds, 2 to being able to independently stand on one leg but unable to maintain the posture for three seconds or more, 3 to being able to independently stand on one leg for 5-10 seconds, and 4 to being able to independently stand on one leg for more than 10 seconds. The worse score marked with the right or left leg was chosen in this task.

Mini-Mental State Examination

This screening examination assesses cognitive functions of elderly patients [14,15]. This examination consists of five variables such as orientation, registration, attention and calculation, recall, and language. The maximum score is 30, and a score of 23 or less suggests cognitive impairment. 

Weight-bearing ratio

WBR is a simple and quantitative evaluation of the lower extremities' motor strength using only two weight scales. This evaluation is considered to be related to ambulation abilities [6,7]. WBR was measured as follows: elderly patients stood on two weight scales positioned 10 cm apart with a 30-degree angle between their heels. They were instructed to shift their weight to one leg and maintain the posture for five seconds. The weight on each leg was recorded, and the WBR was calculated as the average of the maximum weight on each leg divided by the total body weight.

Statistical analysis

Patients were categorized into two groups based on their ambulation aids: bilateral upper extremity support using walkers or wheeled mobility aids and unilateral support using a cane. Data normality was assessed using the Shapiro-Wilk test. Group differences in continuous variables were compared using independent samples t-tests (for normally distributed data) or Mann-Whitney U tests (for non-normally distributed data). Pearson correlation analysis assessed the relationship between BBS scores and WBR. Partial correlation analysis, controlling for age, was also conducted. Statistical significance was set at p < 0.05. All statistical analyses were performed using SPSS Statistics version 24.

## Results

Patient characteristics

35 patients (11 male, 24 female) with a mean age of 81.8 ± 7.4 years (range 67-93 years) were enrolled. The mean body weight was 51.1 ± 10.1 kg (range 31.0-74.7 kg). Five patients received physical therapy for cerebrovascular disease and 30 patients for motor disorders. Pre-admission surgeries included osteoporotic vertebral fractures (n=2), femur fractures (n=28), cerebellar infractions (n=4), and shunt surgery for normal-pressure hydrocephalus (n=1). The mean MMSE score on admission was 25.3 ± 3.8 (range 17-30) (Table 1).

**Table 1:** Patients’ characteristics in this study The table shows the characteristics of the elderly patients in this study. Average ± standard deviation (maximum-minimum). MMSE: Mini-Mental State Examination; FAC: Functional Ambulation Category

Patients number (male:female)	35 (11:24)
Age (years old)	81.8 ± 7.4 (67-93)
Body weight (kg)	51.1 ± 10.1 (31.0-74.7)
Cerebrovascular disease	5 patients
Infraction	4 patients
Normal pressure hydrocephalus	1 patient
Motor disorder	30 patients
Osteoporotic vertebral fractures	2 patients
Femur fractures	28 patients
The MMSE scores on admission	25.3 ± 3.8 (17 -30)
Period of the ambulation evaluated from the onset of the underlying disease (days)	58.9 ± 17.2 (28-101)
FAC 3	8 patients
FAC 4	2 patients
FAC 5	23 patients

Ambulation status

Ambulation status was assessed an average of 58.9 days (range 28-101 days) after disease onset. The distribution of Functional Ambulation Categories (FAC) was as follows: FAC 3 (n=8), FAC 4 (n=2), and FAC 5 (n=23). 20 patients required bilateral upper extremity support (walkers or wheeled mobility aids), while 15 patients ambulated independently using a cane.

Group comparisons

Significant differences were observed between the two ambulation groups (Table 2) for age (p<0.05), WBR (p<0.05), duration of ambulation since disease onset (p<0.05), and BBS scores (p<0.01). No falls occurred during the study period.

**Table 2:** Clinical features according to ambulation status of the patients enrolled in this study The table shows statistical results between the two groups. Average ± standard deviation (minimum-maximum). *: p<0.05 (parametric analysis), **:  p<0.01 (parametric analysis), §: p<0.05 (non-parametric analysis) MMSE: Mini-Mental State Examination; WBR: Weight-bearing ratio; BBS: Berg Balance Scale

	Ambulation with bilateral upper extremities support (20 patients)	Independent ambulation with a stick (15 patients)
Age.** (years old)	85.1 ± 6.4 (71-93)	77.4 ± 6.4 (67-93)
Body weight (kg)	49.7 ± 10.6 (31.0-74.7)	53.0 ± 9.4 (36.0-65.0)
MMSE scores on admission	24.6 ± 3.9 (18-30)	26.3 ± 3.6 (17-30)
Period of the ambulation evaluated from the onset of the underlying disease^§^	54.7 ± 19.0 (28-101)	64.5 ± 13.1 (38-88)
WBR*	0.73 ± 0.11 (0.51-0.91)	0.81 ± 0.12 (0.54-0.98)
The sum scores of BBS**	38.9 ± 5.5 (29-52)	45.9 ± 6.8 (28-56)

Correlation analysis

A strong positive correlation was found between BBS scores and WBR (r=0.824, p<0.01). Negative correlations were observed between age and BBS scores (r=-0.422, p<0.05) and between age and WBR (r=-0.533, p<0.05). The positive correlation between BBS scores and WBR remained significant after controlling for age using partial correlation analysis (r_p_=0.781, p<0.01).

## Discussion

In this study, we evaluated the BBS and WBR in elderly patients undergoing the recovery phase rehabilitation. Enrolling the elderly patients whose ambulation status was independent enough (the FAC of 3 or more), we identified significant differences in the BBS scores and WBR between patients requiring silver cars or pick-up walkers and those ambulating with a stick. A positive correlation was also identified between the BBS and WBR. These results seem to suggest that WBR may be a simple and useful indicator for choosing ambulation-supporting tools for elderly patients undergoing recovery phase rehabilitation.

The BBS evaluates elderly patients’ physical performance and can be a good indicator of safe ambulation without falling. Berg et al. described that a score of 45 could be a cut-off value for safe and independent ambulation in the home without falling accidents [11]. A score of 46 or 49 has also been reported as a potential cut-off value related to falling events in elderly people [16,17]. Shumway-Cook et al. evaluated the physical performance of 44 elderly volunteers aged 65-94 using the BBS. They categorized those volunteers into two groups based on the occurrence of a falling episode within six months. A cut-off value of 49 in the BBS was related to fall risk with 77% sensitivity and 86% specificity [16]. Lajoie et al. enrolled 125 elderly in nursing homes and senior residences. They were classified into two groups: 45 fallers and 80 non-fallers. The BBS scores in fallers were significantly lower than those in non-fallers and had a cut-off value of 46, predicting a fall event in the recruited elders [17]. Although the recruited patients in our study were different from the elders of the previous studies, because of the hospitalization status, the mean BBS in the patients independently ambulating (45.9) seems equivalent to the preceding results. Previously, a moderate or high correlation was identified between the BBS and the following ADL and ambulation measurements, such as the Barthel Index, Dynamic Gait Index, Fugal Meyer Test, and gait speed [18,19]. However, little has been described concerning the correlation between the BBS and WBR [6].

Itotani et al. evaluated the BBS and WBR in hemiplegic stroke patients [6]. They recruited 17 patients (mean age: 61.0 years old) and divided them into two groups: independently ambulating using a cane and lower limb orthosis and requiring a wheelchair. In their study, the results of the BBS and WBR were significantly higher in the former group (53.2 ± 2.6 and 97.7 ± 2.5%, respectively) than in the latter group (40.2 ± 6.4 and 71.3 ± 12.1%, respectively). Our study differs from theirs in that we enrolled patients whose ambulation status corresponded to the FAC of 3 or more. Then, we divided the patients into two groups according to the ambulation independence: ambulation using silver cars or pick-up walkers, or independent ambulation with a stick. The statistical significance was also detected in the BBS and WBR between our two groups. The BBS and WBR results in our study were lower than those in the study of Itotani et al. This is probably because the patients in our study were older than those in their study. In addition, the correlation between the BBS and WBR was not evaluated in their study while a positive correlation between them was identified in our study.

As shown in our study, the BBS scores and WBR might reflect the balanced abilities of elderly patients undergoing recovery phase rehabilitation. Moreover, the WBR could be a simple and adequate measurement in selecting ambulation-supporting tools. To validate the usefulness of the WBR, further research enrolling more patients in various disease phases (i.e., acute, chronic) is warranted. Because the WBR can be assessed in various postures and circumstances, this evaluation can also be approached from this concern [6].

Limitations

Our study has a strength in that the positive correlation between BBS and WBR was identified in elderly patients undergoing recovery phase rehabilitation. This regard has not been often approached previously. Thus, our study provides clinical importance to this field. However, several limitations need to be considered. First, the retrospective design and relatively small sample size from a single recovery-care hospital limit the generalizability of our findings. Further research with larger, more diverse samples is warranted to confirm these results as an external validation. Second, the predominance of patients with orthopedic diseases may restrict the applicability of our findings to patients with other underlying conditions. Third, this study was limited to elderly patients in the recovery phase of rehabilitation. It remains unclear whether similar results would be obtained in patients of different ages or in acute and chronic care settings. Additionally, the patients' cognitive functions evaluated using MMSE ranged from 17 to 30. It may be controversial whether all the patients understood the therapists' indications appropriately, resulting in sufficient evaluation of physical performance.

## Conclusions

This study demonstrates a strong positive correlation between BBS scores and WBR in elderly patients undergoing recovery-phase rehabilitation. These findings suggest that WBR may provide a simple, efficient, and clinically useful alternative or supplementary assessment of ambulation independence. However, further research with larger, more diverse patient populations across different ages and clinical phases is needed to validate these findings and establish the clinical utility of this measure.
